# The brain’s immune code: decoding antigen presentation at the meningeal-glymphatic interface in neurological disorders

**DOI:** 10.3389/fimmu.2026.1960846

**Published:** 2026-09-15

**Authors:** Wanying Li, Yuying Liang, Liwei Li, Hui Sun, Qiang Liu

**Affiliations:** 1 Xiangya School of Medicine, Central South University, Changsha, China; 2 Department of Thoracic Surgery, First Affiliated Hospital of Guangzhou Medical University, Guangzhou, China; 3 School of Pharmacy, College of Medicine, Hunan Normal University, Changsha, China; 4 Department of Neurology, First Affiliated Hospital of Guangzhou Medical University, Guangzhou, China; 5 Department of Neurology, Loudi Hospital of the First Affiliated Hospital of Guangzhou Medical University, Loudi, China

**Keywords:** antigen presentation, glymphatic pathway, immune privilege, meningeal lymphatic vessels, neuroimmunology, neurological disorders

## Abstract

The classical view of central nervous system (CNS) immune privilege has evolved from isolation to regulated crosstalk. Perivascular cerebrospinal fluid-interstitial fluid transport and meningeal lymphatic vessels (mLVs) move brain-derived molecules toward immune-rich border tissues and cervical lymph nodes, where antigen-presenting cells (APCs) decode this molecular drainage into adaptive immunity. We frame this sequence as a brain-immune code, wherein the identity, timing, route, and cellular presenter of exported antigens collectively dictate whether T-cell responses become suppressive, inflammatory, or ineffective. By comparing aging and Alzheimer’s disease, multiple sclerosis, experimental autoimmune encephalomyelitis, and brain tumors, we illustrate why this same interface can mediate proteostatic clearance, pathogenic autoimmunity, or insufficient antitumor immunity. Accordingly, therapeutic strategies should tune drainage and antigen interpretation in a disease-, stage-, and compartment-specific manner, rather than uniformly enhance or suppress lymphatic function. Key priorities include integrated measurement of glymphatic and mLV function, time-resolved immunopeptidome mapping, definition of non-redundant APC roles, and validated human biomarkers linking drainage to immune status.

## 1 Introduction

For much of the twentieth century, the blood-brain barrier (BBB), sparse parenchymal leukocytes, and the apparent absence of conventional lymphatic supported a model of CNS immune isolation. The rediscovery of functional dural lymphatic vessels in 2015, later confirmed in human and non-human primates, replaced that binary view with one of controlled accessibility (1–4). In parallel, work on perivascular cerebrospinal fluid (CSF)-interstitial fluid (ISF) exchange provided a route by which molecules from deep brain tissue could reach immune-active borders (5, 6).

The resulting literature often treats waste clearance and immune surveillance as parallel functions. Yet the amount of material drained cannot by itself explain why impaired mLV function aggravates protein accumulation in Alzheimer’s disease (AD), whereas intact drainage can support pathogenic T-cell responses in experimental autoimmune encephalomyelitis (EAE), or why enhancing the same route can improve antitumor immunity. We therefore use the term brain-immune code to describe the antigenic information made available by CNS outflow and its interpretation by antigen-presenting cells (APCs) and lymphocytes. Our central argument is that neurological disease reflects not simply too little or too much drainage, but a context-dependent failure in how brain-derived antigens are routed, presented, and acted upon. This focused review follows a transport-sampling-presentation-immune fate framework and uses AD, multiple sclerosis (MS)/EAE, and brain tumors as contrasting test cases rather than attempting a catalogue of cerebral lymphatic anatomy or neurological disease.

## 2 From parenchymal transport to meningeal sampling

Upstream antigen availability begins with movement through the brain extracellular space. In the glymphatic working model, CSF enters periarterial spaces, exchanges with ISF, and redistributes interstitial solutes toward perivenous and other efflux routes. Astrocytic aquaporin-4 (AQP4) polarization, arterial pulsatility, neuronal activity, and the patency of perivascular spaces maintained by parenchymal border macrophages can influence this transport (6–8). However, glymphatic flow should not be presented as a universally established, single-direction bulk current. Diffusion, solute-specific transport, and secretion or clearance across the BBB also contribute, and their relative importance varies by molecule, compartment, physiological state, and experimental method (6, 7).

After brain-derived solutes reach CSF and the subarachnoid space, several partly overlapping exits become available. Arachnoid cuff exit (ACE) points around bridging veins create discontinuities through which CSF and molecules can pass between the subarachnoid space and dura (8). Dorsal mLVs near the superior sagittal and transverse sinuses and basal vessels along the skull base then connect intracranial fluid to cervical lymphatic networks. Three-dimensional mapping supports conserved meningeal drainage circuits in mice and humans, while the nasopharyngeal plexus and cribriform routes provide parallel extracranial drainage (9, 10). Their relative contributions remain unsettled: results differ with injection site, tracer, body position, species, and whether fluid, macromolecules, or cells are measured. Transport therefore regulates which antigens become visible at a given border and time, but it does not determine the direction of the immune response. That decision is made within the cellular niches that sample and present the exported material.

## 3 Decoding antigen presentation at brain borders

The dura is an immune-rich tissue rather than a passive covering. Macrophages, dendritic cells (DCs), T cells, B cells, innate-like lymphocytes, and stromal cells are concentrated around dural venous sinuses, where CSF-borne molecules accumulate and blood-derived cells can survey the border (11–13). Myeloid cells can also arrive from adjacent skull bone marrow through specialized channels. This architecture permits two linked forms of surveillance: local capture and presentation of CNS-derived antigens at the meninges, and delivery of soluble antigen or migratory APCs through mLVs to deep cervical lymph nodes (dCLNs).

The dCLNs are not passive recipients of meningeal drainage but rather specialized immunological arenas where CCR7-dependent trafficking delivers both soluble CNS antigens and migratory APCs from the meninges into the T-cell zones, enabling efficient encounter with naive lymphocytes (14); within these nodes, the contextual parameters of antigen identity, APC activation state, co-stimulatory molecule expression, and cytokine milieu jointly instruct the differentiation of T cells toward regulatory, effector, or exhausted fates.

Cribriform plate route as a quantitatively major drainage pathway (15). CSF drains along olfactory nerve sheaths through the cribriform plate into nasal lymphatics, then to cervical lymph nodes—providing a direct conduit for brain-derived antigens to reach peripheral immune organs. This route may be particularly important for clearance of low-molecular-weight solutes and viral antigens, whereas the dural lymphatic route handles larger particulate antigens and immune cells. MHC-II immunopeptidomics has supplied a mechanistic anchor for the brain-immune code. In mice, CNS-enriched self-peptides, including sequences derived from myelin basic protein, were detected on MHC-II molecules in the brain borders and dCLNs. These endogenous regulatory peptides were presented by macrophages and DCs, but not appreciably by B cells, and elicited suppressive CD4+ T-cell responses that constrained neuroinflammation through CTLA-4- and TGF-beta-dependent mechanisms (16). Inflammation changed antigen-processing machinery, reduced presentation of these regulatory peptides, and created an altered self-peptide repertoire. The authors termed the protective sequences guardian peptides. This is a powerful working model, but its full antigen repertoire, cellular sources, and relevance across human CNS compartments remain unresolved; it should not yet be treated as a complete molecular code (6, 16).

APC identity is therefore as important as antigen identity. DCs are specialized for priming naive T cells in lymphoid tissue, whereas border-associated macrophages are abundant local MHC-II-expressing cells and may dominate antigen re-encounter at CNS interfaces (17). B cells can shape a distinct peptide repertoire and, in the meninges, developing B cells can undergo negative selection against CNS antigens (18). Differences in antigen uptake, proteolysis, MHC loading, co-stimulation, and cytokine exposure may direct conventional regulatory T cells, unconventional suppressor CD4+ cells, or inflammatory effector cells. The same peptide presented by a steady-state macrophage and by an inflammation-conditioned DC should therefore not be assumed to carry the same immunological meaning.

The decoding niche also feeds back on drainage. VEGF-C/VEGFR3 signaling supports mLV development and remodeling, whereas CCL21 produced by lymphatic endothelial cells and CCR7 expressed by DCs and lymphocytes regulate cell egress toward cervical nodes (11, 12, 14). Conversely, aging-associated meningeal T-cell accumulation and increased interferon-gamma (IFN-gamma) can impair lymphatic integrity and drainage, while reduced CCR7 expression promotes immune-cell retention (19, 20). Thus, the code is generated by a loop, not a one-way pipe: route and flow determine antigen access; the presenter and inflammatory context determine immune fate; and the resulting immune response remodels the route itself (**Figure 1**).

**Figure 1:**
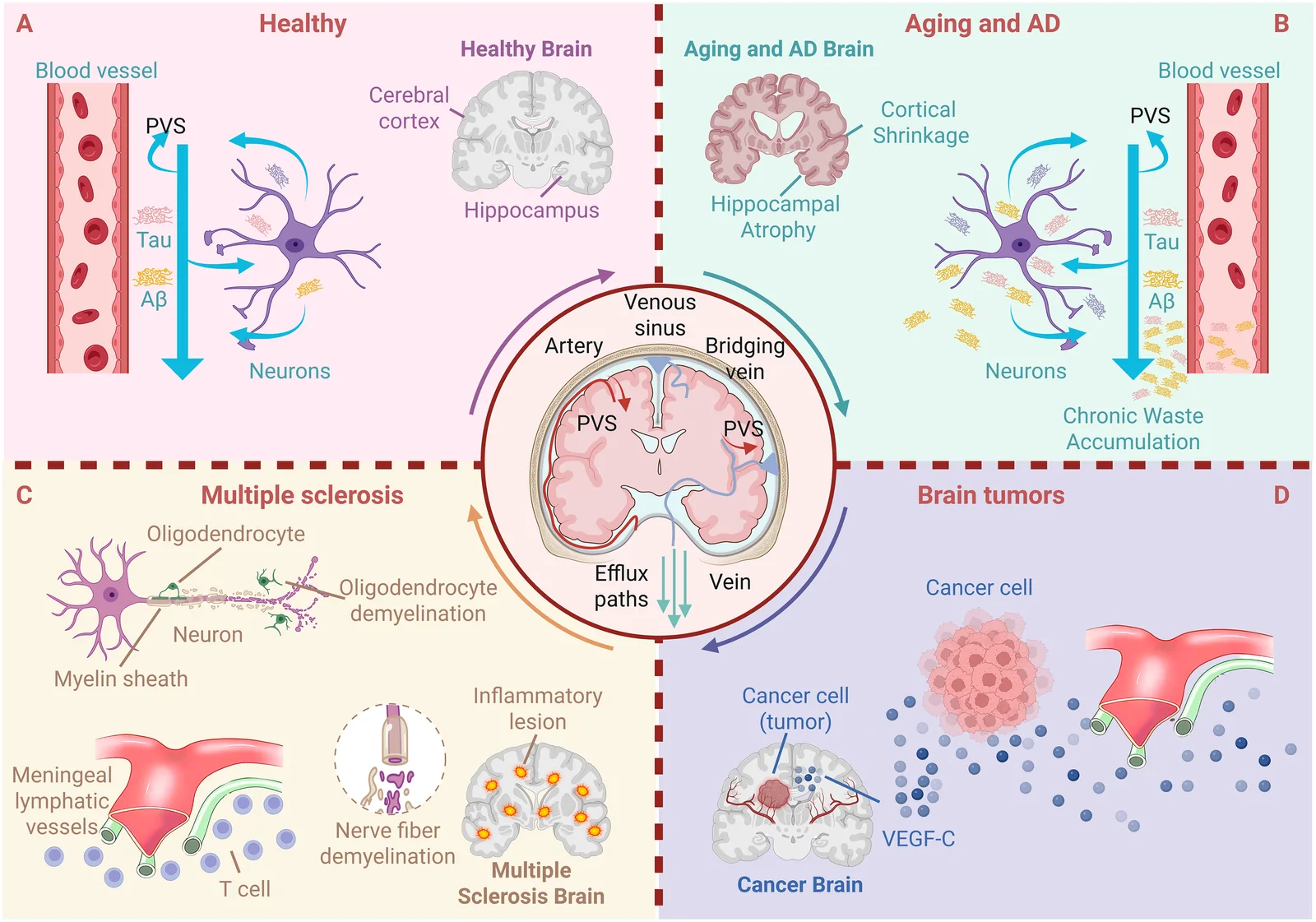
Four-part scientific illustration compares healthy brain, aging and Alzheimer’s disease (AD), multiple sclerosis, and brain tumors. Center shows a brain cross-section highlighting perivascular spaces (PVS) and efflux pathways. Section **A** (top left) depicts normal PVS function with removal of tau and amyloid-beta from neurons in a healthy brain. Section **B** (top right) shows aging and AD brain with cortical shrinkage, hippocampal atrophy, and chronic waste accumulation due to impaired clearance. Section **C** (bottom left) illustrates multiple sclerosis with oligodendrocyte demyelination, inflammatory lesions, and affected meningeal lymphatic vessels. Section **D** (bottom right) shows cancer brain with tumors, spread of cancer cells, and VEGF-C involvement.

## 4 Disease-specific rewiring of the brain-immune code

### 4.1 Aging and Alzheimer’s disease: failed clearance and distorted surveillance

Aging weakens several connected components of CNS clearance, including vascular pulsatility, perivascular AQP4 polarization, glymphatic transport, cervical lymph-node structure, and mLV drainage. In mouse models, photodynamic ablation or other impairment of mLVs reduces CSF outflow, worsens Aβ accumulation and cognition, and shifts microglia toward inflammatory transcriptional states; VEGF-C-mediated restoration can improve drainage and the efficacy of anti-Aβ antibodies (19, 21). Yet direct drainage of every Aβ or tau species through mLVs has not been demonstrated, and one study summarized in the supplied literature found that chronic VEGF-C/VEGF-D trapping altered CSF drainage without changing total Aβ plaque burden (22). Effects may therefore also be mediated indirectly through glymphatic exchange, vascular function, microglia, or peripheral immunity. Immune remodeling adds a second layer. Aged meninges accumulate T and B cells; IFN-γ-producing T cells can reduce mLV integrity, while diminished CCR7-dependent egress favors T-cell retention and is associated with impaired glymphatic function and cognition (19, 20).

Clonally expanded CD8+ T cells have also been detected in the CSF of patients with AD, although their antigen specificity and causal role remain uncertain (23). B cells likewise show protective and detrimental effects across models. The most defensible conclusion is therefore not that a particular lymphocyte is uniformly good or bad, but that drainage failure, antigen retention, and chronic inflammatory presentation can reinforce one another. For AD, the therapeutic direction is to restore subphysiological clearance while correcting the inflammatory context, with effects tested separately in amyloidosis, tauopathy, APOE4, and mixed pathology.

### 4.2 Multiple sclerosis/EAE: productive drainage can support pathogenic priming

MS/EAE provides the clearest counterexample to the assumption that more drainage is always beneficial. CNS antigens and CCR7-dependent immune-cell traffic through meningeal and cribriform lymphatic routes can support activation or reactivation of encephalitogenic T cells in cervical nodes and at brain borders. Ablating mLVs around the time of, or after, EAE induction reduced inflammatory gene programs in brain-reactive T cells and attenuated disease in one experimental framework (14). However, prolonged VEGFR3 blockade that caused marked dural lymphatic regression did not alter autoimmune neuroinflammation in another study (24). Differences in timing, target, route compensation, and EAE protocol probably matter. EAE also incompletely models human MS, and complete blockade could impair homeostatic surveillance, immune-cell egress, or repair.

Antigen quality may further explain this duality. Neuroinflammation reduces presentation of regulatory self-peptides and can modify myelin antigens, potentially weakening suppressive T-cell recognition while favoring immunogenic epitopes (6, 16). The relevant therapeutic question is therefore not a binary choice between enhancing and suppressing mLVs. It is whether pathogenic antigen presentation can be limited during a defined disease phase, or redirected toward antigen-specific tolerance, without disabling beneficial border immunity. Inflammation alters the self-peptide repertoire through altered proteolysis, changed MHC trafficking and peptide loading, differential APC subset recruitment and activation (25).

### 4.3 Brain tumors: inadequate priming within an immunosuppressive context

Brain tumors illustrate the opposite failure: tumor antigens may drain or be presented inefficiently, while glioma-associated myeloid cells, regulatory lymphocytes, checkpoint signaling, and T-cell exhaustion suppress effective immunity (3). In mouse intracranial tumor models, mLVs supported tumor-fluid drainage, DC trafficking, and cervical-node priming. Increasing VEGF-C enhanced CD8+ T-cell priming and tumor infiltration and improved responses to radiotherapy or checkpoint blockade (26, 27). These results establish proof of principle for lymphatic augmentation in neuro-oncology, but they remain preclinical. Tumor-associated lymphatic remodeling is heterogeneous, and lymphatic routes can also facilitate tumor-cell dissemination; safety, tumor type, and treatment timing must therefore be resolved before translation.

Across the three settings, the same interface encodes different outcomes. AD is dominated by insufficient export coupled to chronic immune distortion; MS/EAE can involve efficient presentation of self-antigen in a pro-inflammatory context; brain tumors combine weak or suppressive priming with T-cell dysfunction. The contrast is summarized in **Table 1** and supports a single principle: route activity is therapeutically meaningful only when interpreted together with antigen, APC state, lymphocyte fate, and disease stage.

**Table 1:** Same route, different immune code across neurological contexts.

Context	Dominant route change	Immune decoding	Priority intervention principle
Aging/AD	Reduced CSF-ISF exchange and mLV outflow; Aβ/tau retention	Chronic myeloid activation, T-cell retention and IFN-gamma feedback	Restore drainage toward a physiological range and monitor inflammatory state
MS/EAE	CNS antigen and immune-cell access to cervical nodes and borders	Pro-inflammatory APC-T-cell priming or reactivation; loss of regulatory peptide presentation	Stage-specific restriction of pathogenic presentation or induction of antigen-specific tolerance
Brain tumors	Insufficient or suppressive tumor-antigen drainage and presentation	Weak DC/CD8+ priming, checkpoint signaling and T-cell exhaustion	Enhance antigen delivery with radiotherapy or checkpoint therapy while monitoring dissemination

AD, Alzheimer’s disease; APC, antigen-presenting cell; CSF, cerebrospinal fluid; EAE, experimental autoimmune encephalomyelitis; IFN-gamma, interferon-gamma; ISF, interstitial fluid; mLV, meningeal lymphatic vessel; MS, multiple sclerosis.

## 5 Therapeutic implications: tune, do not simply enhance

Interventions can act on at least three layers. First, they can alter structure and flow. VEGF-C/VEGFR3 manipulation is the best-developed example: in preclinical AD and brain-tumor models it can expand or restore lymphatic function and improve combination therapy (19, 26, 27). Piezo1 agonism, focused ultrasound, photostimulation, and modulation of lymphatic contractility are additional experimental approaches summarized in recent reviews (7, 22). None yet establishes a generally safe human method for chronically increasing CNS lymphatic drainage, and excessive or ectopic lymphangiogenesis could change immune-cell trafficking or tumor spread. Restoring a physiological range is a safer goal than maximizing flow.

Second, therapy can target the decoder rather than the pipe. Candidate strategies include controlling CCL21/CCR7-dependent migration, changing meningeal DC or macrophage co-stimulation, supplementing regulatory self-peptides, and shifting the balance between suppressive and effector T-cell responses (14, 16, 19, 20). Such approaches may permit antigen-specific tolerance in MS or stronger tumor-specific priming without broadly suppressing or activating immunity.

Third, treatment must be stratified by stage and compartment. An AD patient with early clearance failure, an inflammatory MS relapse, and a checkpoint-treated glioblastoma should not receive the same lymphatic intervention. In AD (chronic, progressive), promoting mLV drainage appears beneficial; in acute EAE/MS relapse, the role is more nuanced—VEGF-C therapy may need to be timed to the resolution phase rather than the acute attack phase.

Human translation requires biomarkers that measure more than vessel visibility. Contrast-enhanced and non-contrast MRI can visualize putative meningeal lymphatic networks and associations with cervical nodes, and human aging studies suggest coupled abnormalities in glymphatic and meningeal pathways (1, 28). However, current voxel dimensions exceed the diameter of many mLVs, contrast enhancement can reflect adjacent vascular or dural compartments, and anatomy does not equal transport capacity. An informative biomarker panel should combine fluid kinetics, antigen or proteomic signatures, and immune-cell state, then be validated longitudinally against clinical outcomes.

## 6 Discussion and perspectives

Three controversies define the field. First, the relative contributions of bulk flow, diffusion, BBB transport, and other clearance mechanisms vary across studies; glymphatic transport is a useful model, not an exclusive explanation. Second, the proportion of CSF and solute drainage carried by dorsal or basal mLVs, ACE points, nasopharyngeal/cribriform routes, and spinal pathways remains method- and species-dependent. Third, apparently conflicting disease results may reflect a mistaken effort to assign mLVs a single protective or pathogenic role. Timing is particularly important: altering an established immune response is not equivalent to changing lymphatic development before disease induction.

Several experiments would move the field beyond this binary debate. Glymphatic exchange and mLV drainage should be quantified in the same animal and human studies to identify compensation rather than infer it from separate models. Time-resolved immunopeptidomics should map which CNS antigens appear in the parenchyma, CSF, dura, and dCLNs across disease stages. Conditional manipulation of DCs, border macrophages, lymphatic endothelial cells, and B cells is needed to define non-redundant antigen-processing and presentation roles. Studies should also incorporate age, sex, sleep and circadian state, systemic inflammation, and microbial metabolites, all of which may alter both flow and immune interpretation (3, 6, 12). Most importantly, human longitudinal cohorts must connect imaging-defined transport to cellular and molecular immune readouts rather than extrapolate causal mechanisms from rodents.

The practical goal is precision neuroimmune hydraulics: coordinated control of fluid transport, antigen repertoire, APC state, and lymphocyte fate at the relevant anatomical border and disease phase. This framework preserves the insight behind CNS immune privilege - neural tissue requires unusually tight control of inflammation - while replacing isolation with active, compartmentalized communication. Decoding the brain’s immune code will therefore require moving beyond a binary view of lymphatic function toward a temporally and spatially resolved account of how antigen transport becomes immune fate.
